# A patient-preference cohort study of office versus inpatient uterine polyp treatment for abnormal uterine bleeding

**DOI:** 10.1007/s10397-016-0946-4

**Published:** 2016-05-17

**Authors:** Natalie A. M. Cooper, Lee Middleton, Paul Smith, Elaine Denny, Lynda Stobert, Jane Daniels, T. Justin Clark, on behalf of the OPT trial collaborative group

**Affiliations:** 1 Women’s Health Research Unit, Queen Mary University of London, London, E1 2AT UK; 2 Birmingham Clinical Trials Unit, University of Birmingham, Birmingham, B15 2TT UK; 3 Birmingham Women’s NHS Foundation Trust, Birmingham, B15 2TG UK; 4 School of Clinical and Experimental Medicine, University of Birmingham, Birmingham, B15 2TT UK; 5 Centre for Health and Social Care Research, Birmingham City University, Birmingham, B15 3TN UK; 6 School of Allied and Public Health Professions, Birmingham City University, Birmingham, B15 3TN UK; 7 OPT Trial Office, Birmingham Clinical Trials Unit, College of Medical and Dental Sciences, Robert Aitken Institute for Clinical Research, University of Birmingham, Edgbaston, Birmingham, B15 2TT UK

**Keywords:** Office polypectomy, Abnormal uterine bleeding, Patient preference, Ambulatory gynaecology, Uterine polyp

## Abstract

Uterine polyps can cause abnormal bleeding in women. Conventional practise is to remove them under general anaesthesia but advances in technology have made it possible to perform polypectomy in the office setting. We conducted a patient-preference study to explore women’s preferences for treatment setting and to evaluate the effectiveness and treatment experience of women undergoing uterine polypectomy. Three hundred ninety-nine women with abnormal uterine bleeding who were found to have uterine polyps at diagnostic hysteroscopy were recruited. Office polypectomies were performed in office hysteroscopy clinics, and inpatient procedures were undertaken in operating theatres. Three hundred twenty-four of 399 (81 %) expressed a preference for office treatment. There was no difference found between office treatment and inpatient treatment in terms of alleviating abnormal uterine bleeding as assessed by patients and in improving disease-specific quality of life. Acceptability was lower and patient pain scores were significantly higher in the office group. When offered a choice of treatment setting for uterine polypectomy, patients have a preference for office over inpatient treatment. Ambulatory gynaecology services should be available within healthcare systems to meet patient demand.

## Introduction

Abnormal uterine bleeding affects women of all ages and is the commonest reason for referral to secondary care [1, 2]. Uterine polyps are commonly found in association with abnormal uterine bleeding in both pre- and postmenopausal women [3–7] when investigated with ultrasound or office hysteroscopy. Whilst the risk of occult malignancy within uterine polyps is low, the available evidence supports the current practise of surgically removing uterine polyps to help alleviate bleeding symptoms [8, 9] and this has traditionally been performed under general anaesthesia. However, with advances in endoscopic technology, it is now possible to perform uterine polypectomy under hysteroscopic guidance in an office setting without the need for hospital admission and anaesthesia [10–12]. Furthermore, treatment can be carried out at the same time as diagnosis; the “see & treat” approach [13].

Whilst recruiting to a randomised controlled non-inferiority study which compared office to inpatient polypectomy, we collected data from women who consented to be followed-up, but had a preference for how they were treated and so could not be randomised. We designed this parallel observational study because a pilot RCT to aid the final office polyp treatment (OPT) study design (www.birmingham.ac.uk/research/activity/mds/trials/bctu/trials/womens/opt/index.aspx) had suggested that a substantial proportion of women would exert a preference for treatment setting. We therefore wanted to explore women’s preferences for treatment setting and to evaluate the effectiveness and treatment experience of women when undergoing uterine polypectomy for alleviating abnormal bleeding according to their preference.

## Methods

### Population

All women with abnormal uterine bleeding and a uterine polyp diagnosed at office hysteroscopy [13] were eligible to be recruited into the office polyp treatment (OPT) study. Women in equipoise were recruited to the randomised study [14] and those with a preference for treatment were asked to participate in the preference study as we describe here. Abnormal uterine bleeding included heavy menstrual bleeding, intermenstrual bleeding and postmenopausal bleeding. Women were excluded if office polypectomy was considered not feasible, malignancy was suspected or another surgical uterine intervention was needed. All participants provided written informed consent. In clinics which provided a ‘see and treat’ service, consent was obtained and the patient was registered on the on-line recruitment system prior to the diagnostic hysteroscopy, so that if a uterine polyp was diagnosed and the woman’s preference was for office polypectomy, treatment could be performed straight away without an interruption to register the patient into the study.

### Procedures

Following the diagnostic hysteroscopy, women who agreed to participate in the preference study had their choice of treatment arranged. Those who chose office polypectomy underwent the procedure immediately following diagnosis in most instances, although some participants had their treatment scheduled within the following 8 weeks. Office polypectomies were performed in the office hysteroscopy clinic and inpatient procedures were performed in operating theatres, under general or regional anaesthesia. Office polyp removal was carried out under direct hysteroscopic vision using miniature mechanical (scissors, biopsy cups and grasping forceps) or electrosurgical instruments (bipolar electrodes), with or without the need for minor degrees of cervical dilatation and local anaesthesia (direct cervical infiltration or paracervical injection). Blind avulsion with small polypectomy forceps was also allowed. Women who chose inpatient polypectomy could have traditional dilatation and curettage or removal under vision using a resectoscope. Clinicians were free to choose the operative technique for polypectomy. Endometrial biopsy and medical therapies were permitted when indicated.

### Outcome measures and follow-up

Our main measure of interest was successful treatment, determined by the women’s assessment of their bleeding at 6 months using a dichotomous (success/fail) outcome measure. For women with heavy menstrual bleeding, treatment was considered a success if bleeding had reduced to acceptable levels. For women with intermenstrual or postmenopausal bleeding, the definition was cessation of bleeding.

Other patient reported outcome measures were the women’s subjective assessment of their bleeding using visual-analogue-scales (0 for no bleeding to 100 heaviest imaginable and 0 for no days bleeding to 100 bleeding every day) and response to the question ‘compared to before your treatment, would you say your bleeding is?’ on an ordered Likert scale (much better, little better, same, worse). Health-related-quality-of-life was measured using the generic EuroQol EQ-5D-3L [15] and the disease-specific Menorrhagia Multi-Attribute Scale (MMAS) [16]. All clinical data were collected at baseline and then by mail at 6 months post recruitment.

Patient experience was also evaluated; patients were asked to rate their level of pain 1 h after the procedure and on discharge from hospital using visual-analogue-scales (0 no pain to 100 worst imaginable pain). Women undergoing office polypectomy also rated the level of pain during the procedure. Acceptability of the procedure was assessed using Likert scales and structured questions. This was supplemented by a series of semi-structured qualitative telephone interviews in a purposive sample of women (who had consented to be interviewed) 1 week after the procedure. Rates of successful polyp removal and complications were recorded peri-operatively, and postoperative data were collected for adverse events and need further treatment.

### Statistical analysis

Analyses were performed including all consenting participants in the group of their preference regardless of whether they received their preference, or indeed any, treatment (i.e. intention–to-treat). Chi-squared and *t* tests were used to assess if there were any systematic differences between the preference groups in terms of their baseline characteristics. Odds ratios for successful treatment at 6 month were generated using a logistic regression model (odds ratios were favoured over risk ratios as it is more straightforward to generate stable adjusted estimates [17]. Estimates were adjusted for potential confounders that were considered to be the most clinically important by adding the following 12 variables to the model: predominant bleeding complaint at consent (post-menopausal/heavy menstrual/intermenstrual), site of uterine polyp (fundal/non-fundal), type of uterine polyp (glandular/fibrous), number of polyps (1/2/3+), largest polyp size (continuous variable), grade of surgeon (consultant/less experienced), removal technique (blind/hysteroscopic), detachment technique (electrode/mechanical), age (continuous variable), body mass index (continuous variable), parity (0/1/2/3/4+), and centre of recruitment (Birmingham Women’s Hospital/Royal Hallamshire Hospital Sheffield/other minor centre). A multiple imputation procedure [18] (assumed data was missing at random) was used in this analysis to impute any missing data items. Variables in the imputation model included the outcome variable of interest together with the parameters listed above; 20 imputed data sets were created using the MCMC method with overall estimates and standard error calculated using Rubin’s rules [19]. 95 % confidence intervals were generated along with a *p* value from the associated two-sided chi-squared test. Other binary outcomes and endpoints measured on a continuous scale (scores from MMAS, EQ-5D and VAS scores) were analysed in a similar fashion to the above (linear regression model adjusting for baseline score for continuous variables). Unadjusted estimates are provided for comparison or where an adjustment was not possible because of low group frequencies. No adjustments were made for operative descriptors. Standard tests were used for other outcome measures: paired *t* tests for changes from baseline scores within groups and two-sample *t* tests for continuous data with a normal distribution, Wilcoxon signed rank test for skewed continuous data and chi-squared tests for binary and categorical responses. SAS version 9.2 was used for analyses (PROC MI for the multiple imputation procedure).

## Results

### Recruitment and qualitative assessment of patients’ preference for treatment

**Fig. 1:**
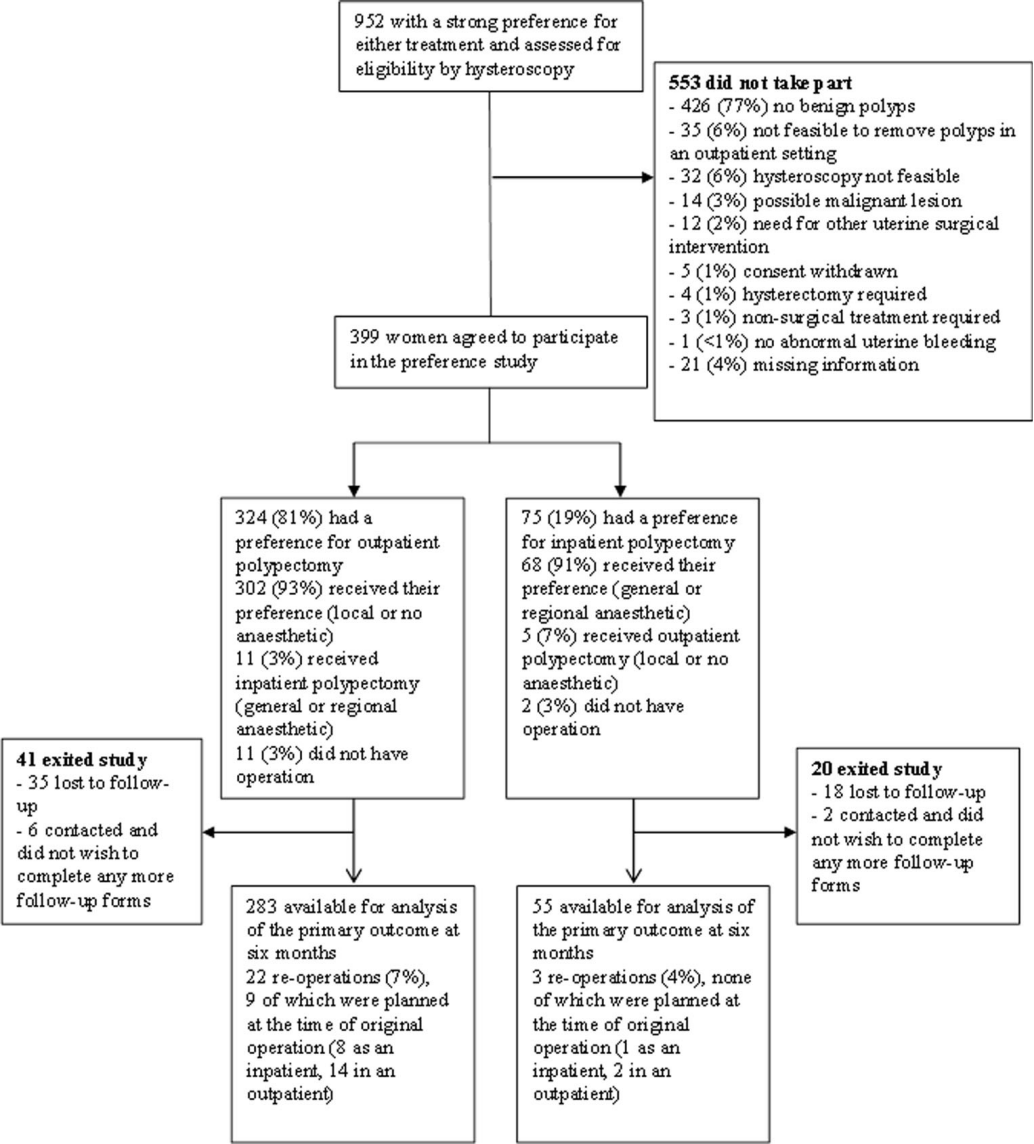
Flow diagram showing enrollment, preference for treatment and follow-up of the study patients

Between April 2008 and July 2011, in 30 UK NHS centres, 952 women with abnormal uterine bleeding and a preference for how they wanted to be treated agreed to participate. Three hundred ninety-nine (42 %) women were recruited with the main reason for ineligibility being no polyp present at diagnostic hysteroscopy. Three hundred twenty-four of 399 (81 %) expressed a preference for office treatment (Fig. 1).

Thirteen women underwent qualitative interviews. Women were asked about their treatment preferences, which were mainly down to individual reasons. Most women choosing office treatment wanted it over and done within one hospital visit, and even though for one of these women the procedure could not be completed she still thought it was the right choice. Of the other women, two had a fear of anaesthetics, one had a pre-existing medical condition, one had children to make arrangements for and one did not want to take time off work. Very few women in the overall preference study chose inpatient treatment and the four interviewed all spoke of a previous bad experience of hysteroscopy or other procedures under local anaesthetic, or embarrassment at being in stirrups, which made them want a general anaesthetic. In both groups of the study, a number of women told of how they had consulted friends and family before attending the clinic, or the nurse in the clinic, in order to make a decision about the procedure, but this had to be weighed up against their personal feelings.

### Participants and follow-up

There were no statistically significant differences between groups in the baseline characteristics of the women (Table 1). Overall, for 48 % (192/399) of the women, the initial complaint was postmenopausal bleeding; 25 % (98/399) had heavy menstrual bleeding and the remaining participants had intermenstrual bleeding, 109/399 (27 %). Three hundred two of 324 (93 %) of the office group received their treatment preference compared with 68/75 (91 %) of the inpatient group (Fig. 1). Sixty-three percent of the women allocated to office polypectomy were treated in ‘see and treat’ clinics. The median time from recruitment to treatment in the office groups and inpatient group were 0 days (IQR = 0, 27) and 31 days (IQR = 7, 55), respectively. Completed primary outcome responses were available from 338/399 (85 %) of participants at 6 months (Fig. 1).

**Table 1:** Baseline characteristics of the patients

		Office polypectomy (*n* = 324)	Inpatient polypectomy (*n* = 75)	*p* value
Age (years)	Mean (SD)	53 (11)	51 (12)	*p* = 0.2
BMI (kg/m^2^)	Mean (SD)	31 (8)^a^	31 (8)^b^	*p* = 0.7
Ethnicity	White	263 (91 %)	54 (93 %)	*p* > 0.9
	Asian	12 (4 %)	2 (3 %)	
	Black	8 (3 %)	1 (2 %)	
	Other	6 (2 %)	1 (2 %)	
	Not given/not known	35	17	
Recruiting centre	BWH^c^	105 (32 %)	24 (32 %)	*p* = 0.5
	RHH^d^	60 (19 %)	18 (24 %)	
	Others^e^	159 (49 %)	33 (44 %)	
Predominant bleeding complaint at randomisation	Post-menopausal^f^	155 (48 %)	37 (49 %)	*p* = 0.2
	Heavy menstrual^g^	75 (23 %)	23 (31 %)	
	Intermenstrual^h^	94 (29 %)	15 (20 %)	
Site of uterine polyp	Fundal	118 (36 %)	25 (33 %)	*p* = 0.6
	Non-fundal	206 (64 %)	50 (67 %)	
Type of uterine polyp	Glandular	229 (71 %)	53 (71 %)	*p* > 0.9
	Fibrous	95 (29 %)	22 (29 %)	
Number of polyps	1	233 (72 %)	58 (77 %)	*p* = 0.6
	2	62 (19 %)	11 (15 %)	
	> = 3	29 (9 %)	6 (8 %)	
Parity	0	49 (15 %)	18 (24 %)	*p* = 0.4
	1	35 (11 %)	6 (8 %)	
	2	121 (37 %)	26 (35 %)	
	3	50 (15 %)	9 (12 %)	
	> = 4	37 (11 %)	8 (11 %)	
	missing	32 (10 %)	8 (11 %)	
Other benign pathology	None	318 (98 %)	74 (99 %)	*p* = 0.8
	SMF/Adhesion/Septum	–	–	
	Adhesion/Septum	–	–	
	SMF	5 (2 %)	1 (1 %)	
	Septum	1 (<1 %)	–	

*SMF* submucosal fibroid

^a^Based on 56 values

^b^Based on 207 values

^c^Birmingham Women’s Hospital

^d^Royal Hallamshire Hospital, Sheffield

^e^28 other centres: median recruitment = 4 (IQR = [2, 9])

^f^16 (10 %) and 3 (8 %) of these women were currently taking a continuous combined ‘no bleed’ HRT in the office and inpatient groups, respectively

^g^Includes one post-menopausal woman (1 %) on a sequential HRT (office group)

^h^Includes two post-menopausal women (2 %) on a sequential HRT (office group)

### Treatment success

There was no significant difference between treatment success in the office and inpatient polypectomy groups with 82 % in each group reporting successful alleviation of bleeding symptoms at 6 months (231/283 versus 45/55, unadjusted OR = 0.99, 95 % CI = 0.47, 2.09; *p* > 0.9; adjusted OR = 1.12, 95 % CI = 0.47, 2.69; *p* = 0.8).

### Operative results

Table 2 details the operative and postoperative results details. Office treatment required less vaginal instrumentation (OR = 0.13, 95 % CI = 0.06, 0.31; *p* < 0.001) and dilatation of the cervix (OR = 0.15, 95 % CI = 0.08, 0.28; *p* < 0.001). Hysteroscopic polypectomy under direct vision was significantly more common (OR = 3.6, 95 % CI = 2.0, 6.4; *p* < 0.001) in the office setting, with electrosurgery being the most popular method of detaching polyps (OR = 2.0, 95 % CI = 1.2, 3.5; *p* = 0.02). Hysteroscopic retrieval of specimens from the uterine cavity was the most common technique in the office setting whereas blind mechanical extraction was preferred in the inpatient group (OR = 5.6, 95 % CI = 3.0, 10.4; *p* < 0.001). The proportion of complete removals was not significantly different between groups (282/312 [90 %] versus 68/73 [93 %], unadjusted OR = 1.4, 95 % CI = 0.5, 3.9; *p* = 0.5; adjusted OR = 1.9, 95 % CI = 0.7, 5.6; *p* = 0.2).

**Table 2:** Operative details and complications

	Office polypectomy	Inpatient polypectomy	Mean difference or OR (95 % CI)^b^, *p* value
Largest polyp size, cm (median [IQR], n)^a^	1.1 [0.8–2.0], 286	1.0 [0.8–2.0], 57	0.0 (−0.2, 0.2), *p* > 0.9
Need for cervical dilation = yes	105/303 (35 %)	52/67 (78 %)	0.15 (0.08, 0.28), *p* < 0.001
Use of vaginal speculum = yes	152/301 (50 %)	53/60 (88 %)	0.13 (0.06, 0.31), *p* < 0.001
Use of local anaesthetic = yes	132/313 (42 %)	2/73 (3 %)	25.9 (6.2, 107), *p* < 0.001
Hysteroscopic removal = yes (vs. blind)	246/299 (82 %)	36/64 (56 %)	3.6 (2.0, 6.4), *p* < 0.001
Scope diameter (mm)	4.0 [4.0–6.0], 201	5.5 [4.0–6.0], 42	−1.0 (−1.0, −1.0), *p* < 0.001
Method used to detach	*n* = *287*	*n = 65*	2.0 (1.2, 3.5)^c^, *p* = 0.02
Electrode	155 (54 %)	24 (37 %)	
Mechanical	102 (36 %)	35 (54 %)	
Combination	30 (10 %)	6 (9 %)	
Method of retrieval	*n = 292*	*n = 62*	5.6 (3.0, 10.4)^d^, *p* < 0.001
Hysteroscopic	193 (66 %)	16 (26 %)	
Mechanical	69 (24 %)	43 (69 %)	
Combination	11 (4 %)	–	
None	19 (7 %)	3 (5 %)	
Surgeon grade = consultant	233/305 (76 %)	40/67 (60 %)	2.2 (1.3, 3.8), *p* = 0.005
Time taken for polypectomy, min (median [IQR], n)	10 [5–15], 290	10 [7–15], 52	−1.5 (3.0, 0.0), *p* = 0.3
Time in office room/theatre, min (median [IQR], n)	30 [20–35], 285	33 [25–45], 53	−6.0 (−10.0, −2.0), *p* = 0.003
Removal success	*n = 312*	*n = 73*	1.4 (0.5, 3.9)^f^, *p* = 0.5
Complete	282 (90 %)	68 (93 %)	
Partial^e^	22 (7 %)	3 (4 %)	
Failed^e^	8 (3 %)	2 (3 %)	
Operative complications	*n = 302*	*n = 67*	
Vaso-vagal episode	17 (6 %)	–	
Patient discomfort	9 (3 %)	–	
Cervical trauma	1 (<1 %)	1 (1 %)	
Uterine perforation	–	–	
Other^g^	1 (<1 %)	–	
Postoperative complications	*n = 301*	*n = 67*	
Vaso-vagal episode	14 (5 %)	2 (3 %)	
Vomiting	3 (1 %)	2 (3 %)	
Severe pain	–	2 (3 %)	
Further treatment/procedure given	*n = 292*	*n = 64*	
Mirena IUS	42 (14 %)	8 (13 %)	
Tranexamic acid	9 (3 %)	–	
Progestogens	3 (1 %)	–	
Endometrial destruction	2 (1 %)	–	
Local oestrogen cream	2 (1 %)	–	
Mefenamic acid	1 (<1 %)	1 (2 %)	
Contraceptive pill	1 (<1 %)	–	
Missing treatment name	2 (1 %)	1 (2 %)	

Numbers in italics refer to the responses received for that particular question

n = number of responses

^a^Polyp size was estimated hysteroscopically

^b^Mean difference < 0 indicates lower with office, similarly OR < 1 is lower with office. For skewed variables presented with medians, differences in location between groups were calculated using Hodges-Lehmann estimates and Moses’ confidence intervals

^c^Odds ratio calculated from ‘electrode’ versus any other category

^d^Odds ratio calculated from ‘hysteroscopic’ versus any other category

^e^Nine (3 %) partial or failed patients in the office group and none in the inpatient group were immediately scheduled for reoperation. Six of these were scheduled to be an inpatient. *Partial or failed reasons in the office group* (%’s given of the total number, 312): patient discomfort (9, 3 %), unable to locate blindly (5, 2 %), unable to access under vision (4, 1 %), polyp too large (3, 1 %), failed hysteroscopy (1, <1 %), base cut but unable to remove (1, <1 %), wide base unable to fully resect (1, <1 %), vaso-vagal episode (1, <1 %), difficult access to base of polyp (1, <1 %), missing reason (4, 1 %); *partial or failed reasons in the inpatient group* (%’s given of the total number, 73): unable to access under vision (1, 1 %), unable to locate blindly (1, 1 %), deep sub-mucous fibroid polyp (1, 1 %), too broad base (1, 1 %), missing reason (1, 1 %)

^f^Odds ratio calculated from ‘partial’ or ‘failed’ versus complete

^g^Other complications: nausea

### Serious adverse events

No serious adverse events occurred in the preference study. The most common perioperative complications in the office group were induced vaso-vagal reactions affecting 6 % of the cohort. Vaso-vagal reactions also occurred postoperatively with a similar percentage in each cohort (5 % of the office group versus 3 % of the inpatient group) (Table 2).

### Quality of life and bleeding scores

Condition-specific quality of life and bleeding scores were significantly improved from baseline at 6 months in both groups with no significant differences between them (Table 3). Generic quality of life scores (Euroqol EQ-5D) were improved from baseline in the office group but not in the inpatient group with no difference between the groups at 6 months. The non-significant increase within the inpatient group may be due to the relatively small number of women in this group.

**Table 3:** Results of quality of life assessments, bleeding and pain scores and procedure acceptability

	Office polypectomy	Inpatient polypectomy		
	Mean (SD, *n*)	Mean (SD, *n*)	Difference (95 % CI)^a^, *p* value	Adjusted difference^h^ (95 % CI), *p* value
MMAS^b^				
Baseline	63 (26, 163)	61 (28, 37)		
6 months	77 (25, 135)^g^	79 (25, 25)^g^	−3 (−12, 7), *p* = 0.55	−4 (−14, 3), *p* = 0.18
EuroQol EQ-5D^c^				
Baseline	0.79 (0.26, 312)	0.72 (0.30, 71)		
6 months	0.82 (0.25, 289)^g^	0.81 (0.30, 56)	0.00 (−0.07, 0.06), *p* = 0.88	0.01 (−0.05, 0.08), *p* = 0.64
EuroQol health thermometer^d^				
Baseline	78 (18, 305)	75 (21, 71)		
6 months	78 (19, 291)	79 (20, 57)	−2 (−7, 3), *p* = 0.34	−1 (−4, 5), *p* = 0.81
Bleeding duration visual analogue scale^e^				
Baseline	39 (26, 74)	38 (26, 23)		
6 months	30 (28, 65)^g^	18 (19, 16)^g^	−13 (−27, 2), *p* = 0.09	−13 (−27, 2), *p* = 0.09
Bleeding amount visual analogue scale^f^				
Baseline	59 (28, 75)	58 (26, 23)		
6 months	32 (28, 68)^g^	26 (27, 16)^g^	−4 (−19, 11), *p* = 0.61	−3 (−20, 14), *p* = 0.72
Operation pain scores				
During procedure^i^	42 (26, 296)	–	–	–
60 min after procedure^**i**^	27 (24, 247)	20 (24, 60)	−7 (−14, 0), *p* = 0.04	−8 (−11, −4), *p* = 0.03
On discharge^**i**^	22 (21, 276)	13 (18, 57)	−9 (−15, −3), *p* = 0.003	−9 (−15, −3), *p* = 0.002
	*n* (%)	*n* (%)	OR (95 % CI), *p* value^j^	Adjusted OR^h^ (95 % CI), *p* value
Operation acceptability				
Totally	194 (65 %)	52 (81 %)		
Generally	48 (16 %)	9 (14 %)	0.21 (0.06, 0.69), *p* = 0.01^k^	0.19 (0.05, 0.70), *p* = 0.01^k^
Fairly	50 (17 %)	3 (5%)		
Unacceptable	7 (2 %)	0 (–)		
Exposure embarrassing?				
Extremely	5 (2 %)	2 (3 %)		
Moderately	30 (10 %)	7 (12 %)	1.4 (0.6, 3.0), *p* = 0.46^l^	1.4 (0.6, 3.7), *p* = 0.36^l^
A little	90 (30 %)	8 (13 %)		
No	177 (59 %)	43 (72 %)		
Recommend to a friend?				
Yes/total	282/302 (93 %)	62/64 (97 %)	0.45 (0.10, 2.0), *p* = 0.28	Not possible to compute
Same treatment again?				
Yes/total	283/300 (94 %)	62/63 (98 %)	0.27 (0.04, 2.1), *p* = 0.21	Not possible to compute
Preferred alternative treatment?				
Yes/total	36/299 (12 %)	10/63 (16 %)	1.4 (0.7, 3.0), *p* = 0.41	1.5 (0.7, 3.5), *p* = 0.32

*n* number of responses

^a^Difference between groups at each time point adjusted for baseline score. Estimates of differences >0 favour office polypectomy, those <0 favour inpatient polypectomy

^b^Menorrhagia Multi-Attribute Scale questionnaire. Scores range from 0 (severely affected) to 100 (not affected). Restricted to those with heavy menstrual and intermenstrual bleeding only

^c^Health-related quality of life questionnaire. Scores range from −0.59 (health state worse than death) to 1.0 (perfect health state)

^d^Health-related quality of life questionnaire. Scores range 0 (worst imaginable health state) to 1.0 (best imaginable health state)

^e^Visual Analogue Scale score. Scores range from 0 (no days of bleeding in the last month) to 100 (bleeding every day in the last month). Restricted to those with heavy menstrual bleeding only

^f^Visual Analogue Scale score. Scores range from 0 (no bleeding in the last month) to 100 (heaviest imaginable bleeding in the last month). Restricted to those with heavy menstrual bleeding only

^g^ *p* < 0.05 when compared with baseline score within group (by paired *t* test)

^h^See statistical methods section for details on adjustments

iVisual Analogue Scale score. Scores range from 0 (no pain at all) to 100 (worst imaginable pain). *T* test used for analysis

^j^Estimates of OR > 1 favour office polypectomy, those <1 favour inpatient polypectomy

^k^Totally acceptable/generally acceptable vs. fairly acceptable/unacceptable combined categories used to calculate odds ratio

^l^Extremely/moderately vs. a little/no combined categories used to calculate odds ratio

### Procedure acceptability

Mean pain scores were significantly higher in the office polypectomy group compared with the inpatient group at 1-h post procedure and on discharge (Table 3). Two percent (7/299) of women in the office group compared with no women in the inpatient group felt that the procedure they underwent was ‘unacceptable’. There was no difference between groups in terms of the number of women who would recommend the procedure to a friend, choose to have the same procedure again or in retrospect would have preferred the alternative treatment. In qualitative interviews, the preference patients reported less pain than the randomised ones, using descriptions such as ‘uncomfortable’, ‘bearable’ and ‘better than expected’. This group also reported little postoperative pain describing it like ‘wind’ or ‘period pain’. They balanced pain, which they mainly experienced short term, with the convenience of a fast response to their problem.

### Additional treatments

There was no difference between groups in the number of women using additional medical treatments for their bleeding, or consulting a healthcare provider during the 6-month follow-up period. Twenty-two women in the office group (7 %) and three in the inpatient group (4 %) had at least one further polyp removal (OR = 1.75, 95 % CI = 0.51, 6.00; *p* = 0.4). The total number of women undergoing subsequent gynaecological operations other than polyp removal was higher with office polypectomy (27 [8 %] versus 3 [4 %]) but this difference was not statistically significant (OR = 1.86, 95 % CI = 0.55, 6.35; *p* = 0.3). These operations comprised the following in the office versus inpatient groups, respectively: hysteroscopy, 9 (3 %) versus 2 (3 %); hysterectomy, 9 (3 %) versus 1 (2 %) and endometrial ablation, 3 (1 %) versus 0. The other six operations were in the office group: five cyst removals (2 %) and one ovary removal (<1 %).

## Discussion

The results of this study demonstrate that women with abnormal uterine bleeding and uterine polyps, who have a preference for how they are treated, are more likely to choose office polypectomy than an inpatient procedure. At 6 months, we found no differences between office and inpatient treatment in terms of treating abnormal uterine bleeding and over 80 % women reported successful alleviation of symptoms, regardless of initial bleeding complaint. The duration and amount of bleeding were significantly reduced following both office and inpatient treatment, and no differences were identified according to treatment preference. Similarly, a non-differential but significant improvement in disease-specific quality of life was seen following polypectomy, although generic quality of life appeared only marginally improved.

There was no increased risk of partial or failed polypectomy in the office group in the preference study which is contradictory to the results of the randomised OPT study [14] which found that office treatment was more likely to fail. This is likely to be due to unknown confounders and the small sample size in the inpatient group. Overall pain scores were still significantly higher in the office group than in the inpatient group but these differences were small and so their clinical significance is debatable; differences between groups in the amount of postoperative medication may also have affected this to an unknown degree. Only 2 % of office patients felt that the procedure was unacceptable; findings that are consistent with the concomitant RCT. Not all women underwent their preferred treatment. We can hypothesise that women may have changed their mind about which treatment they wanted but we are unable to provide data as the reasons were not recorded.

In the qualitative interviews, women who had a preference for office treatment appeared to report less pain than those who were randomised to the office setting. Although the number of women who were interviewed was small, this may suggest that women who choose to have the office treatment are more motivated to tolerate and complete the procedure, which may also contribute to the reduced number of failed procedures in the office setting.

The strengths of this study include its size, the multicentre design, a population representative of the UK demographic, the relatively low rates of loss to follow-up and the tailoring of assessment of outcomes to the primary complaint. As this was a preference study, selection bias is an obvious limitation to the interpretation of the results. However, statistical adjustments were made for the obvious confounding factors including type of bleeding, surgical experience, removal method and the site, nature, size and number of uterine polyps.

In comparison to data from the parallel randomised controlled trial [14], the overall level of failed polypectomy in both groups was lower (9 versus 13 %; OR = 0.64, 95 % CI = 0.42 to 0.99; *p* = 0.05) and treatment success higher (82 versus 76 %; OR = 1.4, 95 % CI = 1.0 to 2.0; *p* = 0.06). These findings are unlikely to be due to differences in demographics of the two studies as they were largely the same apart from a slightly older population in the preference study (2.4 years, 95 % CI = 0.9 to 3.7). It is more likely to reflect the selection of more favourable, better motivated women and technically ‘less challenging’ polyps to surgically remove in the office setting by the operating surgeon. However, the effect of counselling by individual surgeons on patient preference for treatment setting or indeed participation in the parallel randomised trial is unclear.

The 4:1 preference for office over inpatient polypectomy resulted in a smaller inpatient cohort and therefore the result estimates are less precise. Additional limitations of our preference study include varying practise between clinicians and a small number of participants failing to get their chosen treatment.

Office polyp treatment appears to be safe, feasible, acceptable and effective for the treatment of abnormal uterine bleeding in women expressing a preference for such treatment. Although this treatment is becoming more widespread, it is not universally available within national healthcare systems. This study demonstrates that when office treatment is available, most women recognise the potential benefits of office treatment and approximately 80 % would choose it over inpatient treatment. In addition, this cohort study and the RCT have both shown that removing uterine polyps appears to alleviate abnormal uterine bleeding, for which data were previously lacking. Thus, there is evidence on both clinical and patient preference grounds, to support prioritising the provision of ambulatory gynaecology services such as office hysteroscopic polypectomy. An important caveat, however, is that women should be informed that office treatment may be associated with more pain and reduced acceptability compared with scheduled inpatient approaches to treatment under general anaesthesia, although they can be reassured that at least eight out of ten women find the procedure to be totally or generally acceptable. Although the preference study did not demonstrate an increased failure rate in the office group, this is likely to be due to confounding factors and the small inpatient group and as the RCT showed that failure was more likely, women should be informed of this, to allow them to make an informed decision about treatment setting. Future research should be directed at evaluating the influence of clinical characteristics and surgical technologies on the feasibility, acceptability and effectiveness of office hysteroscopic polypectomy.

This large, controlled observational study has shown that the majority of women expressing a treatment preference choose to have office treatment of uterine polyps associated with abnormal uterine bleeding. The findings of the study are subject to selection bias; however, they are consistent with the robust data derived from the parallel RCT. The findings, in particular the high levels of patient preference for office treatment, support the conclusions of the RCT that ‘ambulatory’ gynaecological therapeutic services should be made more widely available within healthcare systems. Currently, some women are being denied a choice of treatment setting and consequently are being subjected to the inconvenience and greater burden of inpatient hospital treatment, which if offered an alternative office treatment option, they could avoid.
